# Transfer learning radiomics based on multimodal ultrasound imaging for staging liver fibrosis

**DOI:** 10.1007/s00330-019-06595-w

**Published:** 2020-01-21

**Authors:** Li-Yun Xue, Zhuo-Yun Jiang, Tian-Tian Fu, Qing-Min Wang, Yu-Li Zhu, Meng Dai, Wen-Ping Wang, Jin-Hua Yu, Hong Ding

**Affiliations:** 1 Department of Ultrasound, Zhongshan Hospital, Fudan University, No. 180 Fenglin Road, Xuhui District, Shanghai, 200032 China; 2 Department of Electronic Engineering, Fudan University, No. 220, Handan Road, Yangpu District, Shanghai, 200433 China; 3 Shanghai Institute of Medical Imaging, No. 180 Fenglin Road, Xuhui District, Shanghai, 200032 China

**Keywords:** Liver cirrhosis, Deep learning, Elasticity imaging techniques, Hepatitis B

## Abstract

**Objectives:**

To propose a transfer learning (TL) radiomics model that efficiently combines the information from gray scale and elastogram ultrasound images for accurate liver fibrosis grading.

**Methods:**

Totally 466 patients undergoing partial hepatectomy were enrolled, including 401 with chronic hepatitis B and 65 without fibrosis pathologically. All patients received elastography and got liver stiffness measurement (LSM) 2–3 days before surgery. We proposed a deep convolutional neural network by TL to analyze images of gray scale modality (GM) and elastogram modality (EM). The TL process was used for liver fibrosis classification by Inception-V3 network which pretrained on ImageNet. The diagnostic performance of TL and non-TL was compared. The value of single modalities, including GM and EM alone, and multimodalities, including GM + LSM and GM + EM, was evaluated and compared with that of LSM and serological indexes. Receiver operating characteristic curve analysis was performed to calculate the optimal area under the curve (AUC) for classifying fibrosis of S4, ≥ S3, and ≥ S2.

**Results:**

TL in GM and EM demonstrated higher diagnostic accuracy than non-TL, with significantly higher AUCs (all *p* < .01). Single-modal GM and EM both performed better than LSM and serum indexes (all *p* < .001). Multimodal GM + EM was the most accurate prediction model (AUCs are 0.950, 0.932, and 0.930 for classifying S4, ≥ S3, and ≥ S2, respectively) compared with GM + LSM, GM and EM alone, LSM, and biomarkers (all *p* < .05).

**Conclusions:**

Liver fibrosis can be staged by a transfer learning modal based on the combination of gray scale and elastogram ultrasound images, with excellent performance.

**Key Points:**

*• Transfer learning consists in applying to a specific deep learning algorithm that pretrained on another relevant problem, expected to reduce the risk of overfitting due to insufficient medical images.*

*• Liver fibrosis can be staged by transfer learning radiomics with excellent performance.*

*• The most accurate prediction model of transfer learning by Inception-V3 network is the combination of gray scale and elastogram ultrasound images.*

**Electronic supplementary material:**

The online version of this article (10.1007/s00330-019-06595-w) contains supplementary material, which is available to authorized users.

## Introduction

The multiple causes of chronic liver disease (CLD) follow a common pathway of progressive liver fibrosis, ultimately culminating in cirrhosis. It has been proved that liver fibrosis and early cirrhosis are partly reversible [1]. Hence, an accurate diagnosis of liver fibrosis is essential for the management and determination of the prognosis of patient with CLD. Traditionally, liver biopsy is the reference for assessing hepatic fibrosis. However, it is invasive and painful and has limitations in accuracy influenced by sampling error and intra- and interobserver variability [2–5]. Given these limitations, liver biopsy is not an ideal method for the repeated assessment of disease progression.

Recently ultrasound elastography has been widely used to evaluate the degree of CLD [6]. The shear wave–based elastographic methods mainly include transient elastography, point shear wave elastography, and two-dimensional shear wave elastography (2D SWE), with good intra- and intersonographer reproducibility [7, 8]. 2D SWE quantitatively estimates the tissue stiffness and provides a more accurate correlation of liver elasticity with liver fibrosis stages compared with transient elastography, virtual touch tissue quatification, and serum liver fibrosis indexes [9]. However, liver stiffness measurement (LSM) by 2D SWE can be affected by many factors, such as the operator experience, obesity, the level of transaminases, and the degree of steatosis and necroinflammatory activity [9–12]. The thresholds of 2D SWE for identifying fibrosis stages in patients with chronic hepatitis B (CHB) have shown great variability in previous studies [1, 9, 10, 13]. Therefore, using 2D SWE values alone is likely to be insufficient for accurately assessing liver fibrosis stages.

According to previous studies, radiomics has great potential for the classification of liver fibrosis. Gao et al [14] used texture analysis to classify ultrasound liver images, and the classification accuracies of S0–S4 were 100%, 90%, 70%, 90%, and 100%, respectively. Kayaaltı et al [15] used determine liver fibrosis stage by analyzing some texture features of liver CT images. Acharya et al [16] used the kernel discriminant analysis and analysis of variance techniques to classify images into various stages of liver fibrosis. Yeh et al [17] extracted image features from gray level concurrence and non-separable wavelet transform to classify fibrosis with support vector machine. There are various kinds of traditional methods for calculating features, but they cannot guarantee the completeness of the feature extraction. Recently, deep learning methods have also been used to evaluate liver fibrosis. For example, Wang et al [18] designed four convolutional layers and applied a fully connected layer for the binary liver fibrosis classification. Lee et al [19] developed a deep convolutional neural network and trained four-class model (F0 vs. F1 vs. F23 vs. F4) for predicting METAVIR scores using B-mode ultrasonography images. For deep learning to be successful, it is necessary to use a large training dataset. However, in clinical applications, access to a large number of medical images is difficult and expensive. One pathway to address the issue is the use of transfer learning to improve the performance by transferring knowledge from another domains to the medical US domain [20]. Yu et al [21] investigated the rats fibrosis scoring by transfer learning with AlexNet and compared them against conventional non-deep learning-based algorithms.

In this study, we used transfer learning to analyze elastogram modality (EM) and gray scale modality (GM) and compared the results with the pathological diagnosis of liver fibrosis stage. Comprehensive utilization of the high-throughput information of gray scale and elastogram images would improve the accuracy of liver fibrosis diagnosis. Transfer learning is expected to solve the overfitting problems for medical imaging caused by insufficient medical images.

## Materials and methods

### Patients

The retrospective study was approved by the institutional ethics committee, and informed patient consent was obtained from all patients. Between January 2016 and December 2016, 717 consecutive patients with local liver lesions treated by partial hepatectomy in our hospital were recruited. The inclusion criteria were (*a*) undergoing 2D SWE with the Aixplorer system within two weeks before surgery and (*b*) age 18 years or older. The exclusion criteria were (*a*) patients with a maximum tumor diameter larger than 5 cm, (*b*) 2D SWE technical failures because of obesity, ascites, or tumor located in segment 5 or 6 of the liver, (*c*) patients with a hepatitis virus infection other than CHB, (*d*) antiviral treatment within six months, (*e*) previous liver transplantation, (*f*) intrahepatic cholangiectasis caused by tumor compression or portal thrombosis diagnosed by US or CT/MRI, and (*g*) patients with congestive heart disease. Finally, 466 patients were enrolled in the study; 364 patients were assigned to the training cohort with randomization, and the other 102 patients were enrolled in the test cohort.

### Multimodal ultrasound images

The Aixplorer (SuperSonic Imagine) system was used to obtain images with a convex probe (SC6–1) within 3 days before hepatic surgery. Measurements were performed in the right lobe of the liver through the intercostal spaces. The patients maintained an overnight fast before examination. The US imaging settings including the depth, overall gain, time gain compensation, and compression were optimized. In the elastography examination, the maximum color scale of elastogram was set as 40 kPa. All gray scale and elastogram imaging settings remained constant in all patients. Assisted by a real-time gray scale US image, the ROI was positioned 1–2 cm under the liver capsule and at least 2 cm from lesion margin, avoiding large blood vessels and acoustic shadowing. Once a color map with complete and homogeneous filling was obtained in the SWE box, a Q-box (mean diameter, 20 mm) was used to obtain the LSM. The mean value of the five LSMs was used as the representative measurement of each patient [10].

### Serological examination

Serological examinations were performed after an overnight fast within 1 week before surgery. The platelet count, aspartate aminotransferase, alanine aminotransferase, albumin, gamma-glutamyl transpeptidase, total cholesterol, total bile acid levels, and the international normalized ratio were recorded. The non-invasive serum liver fibrosis indexes—APRI and FIB-4—were determined according to the published formulas [22, 23].

### Pathological examination

Surgical specimens of the focal liver lesions and the adjacent liver tissue were fixed with 10% formalin and routinely embedded in paraffin. Tissue slices of the background liver 0.5–2.0 cm from the lesion periphery were processed with hematoxylin-eosin, Masson trichrome, and reticular fiber staining. Fibrosis was staged according to the Scheuer scoring system, including stages 0, 1, 2, 3, and 4 [24, 25]. All specimens were analyzed by a pathologist with 10 years of experience.

### Transfer learning

**Fig. 1:**
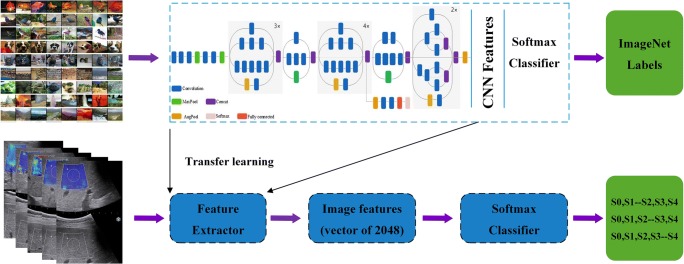
Illustration of the overall transfer learning framework of this study. All the convolutional and pooling layers except the last multinomial logistic classification layer of the Inception-V3 model were taken out as the feature extractor of this study

Transfer learning is to migrate a network trained on a large data set to a different related task, and in this way avoid overfitting problems caused by insufficient training data in regular deep learning. The TL model used in this paper was the Inception-V3 network [26] which was pretrained on ImageNet [27]. The Inception-V3 network employs some inception modules, so it is able to learn both low-level and high-level features with difference convolution kernels. Figure 1 describes our proposed workflow for Inception-V3 (detailed in supplementary). Our medical dataset is smaller by size but different in content compared to the ImageNet. Therefore, we initialized network weights that were fine-tuned on ImageNet and used a binary layer to classify the gray scale and elastogram ultrasound images in the dataset. Meanwhile, we fine-tuned higher-level layers of the network, since that these layers of the network become progressively more specific to the subtle features. To prevent overfitting of the networks to our limited training dataset, we also artificially augmented the training size by random cropping and flipping.

**Fig. 2:**
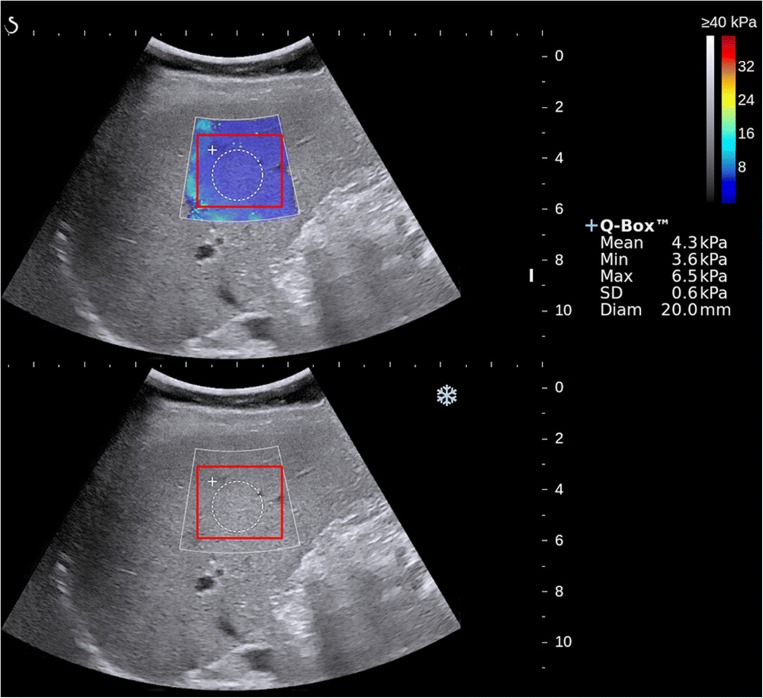
Illustration of the 2D SWE measurement and the ROI of transfer learning (TL) in this study. Image of elastogram image (top), gray scale image (bottom), liver stiffness measurement with Q-Box (white circle area), and ROI of TL (red square area)

In the elastogram image, a square ROI was drawn as large as possible within the color-coded trapezoidal box, containing the Q-box inside. Then the same ROI was automatically generated in the gray scale image below (Fig. 2). For the gray scale ROI, although the image looks gray, it is still an RGB image. The details of the TL model was described in the supplementary method part. The non-transfer learning (non-TL) model was also trained to illustrate the merits of the transfer learning strategy in the liver fibrosis staging.

### Multimodalities

Numerous studies have shown that 2D SWE has excellent diagnostic accuracy, and LSM has a good correlation with the pathological fibrosis stage [11, 28]. The diagnostic value of combining the results of gray scale image analysis and 2D SWE was analyzed in this paper. The confidence coefficient of one image reflected its classification accuracy. After obtaining the confidence of the 2D-SWE (detailed in supplementary), we combined it with the confidence of the gray scale image as features input by logistic regression. We then implemented mini-batch gradient descent to find optimal parameter for classification.

Both the gray scale image and the elastogram contain diagnostic information relating to liver fibrosis. Therefore, after extracting 2048-dimensional features from GM model and EM model trained in previous single-mode experiments, we concatenated features of the two modalities into 4096-dimensional features and used 3 fully connected layers as a classifier. Our proposed workflow for the GM + EM was described in supplementary method.

### Statistical analysis

Descriptive statistics were summarized as mean ± standard deviation (SD) or median and interquartile range (IQR). Comparisons between quantitative variables were made with the *t* test or Mann-Whitney *U* test, and categorical variables were compared using the chi-squared test or Fisher’s test. The area under the receiver operating characteristic curve (AUC) was used as an accuracy index for evaluating diagnostic performance. Differences between AUCs were compared using a Delong test. The sensitivity, specificity, positive predictive value (PPV), negative predictive value (NPV), and positive and negative diagnostic likelihood ratio (LR+, LR−) were calculated. The statistical analyses were performed using SPSS software V.22.0 (IBM Corp.), and MedCalc software V.11.2 (MedCalc Software bvba). Statistical significance level was set as *p* < .05.

## Results

### Patient characteristics

A total of 466 patients were enrolled in the study, including 364 patients with 1820 2D SWE images assigned to the training cohort with randomization, and 102 patients with 510 2D SWE images assigned to the time-independent test cohort to evaluate the diagnostic performance of the developed model. Among the 466 patients, there were 401 CHB-infected patients and 65 patients without CHB infection proved to be S0 by hepatectomy histopathology. The baseline characteristics of the two cohorts were summarized in Table 1. There were no significant differences in either the baseline characteristics or the distribution of patients among the fibrosis stages between the two cohorts (all *p* > .05).

**Table 1:** Patient characteristics between the training cohort and test cohort

Characteristic	Training cohort	Test cohort	*p* value
Number of patients	364	102	/
Number of malignant tumors	317 (87.1%)	92 (90.2%)	.40
Age (year)^†^	54.6 ± 12.2	54.4 ± 12.1	.59
Number of men/women	281/83	72/30	.17
ALT (U/L)^‡^	24 (17–38)	28 (17–39.5)	.42
AST (U/L)^‡^	24.5 (20–35)	25 (20–35.5)	.71
ALB (g/L)^‡^	42 (39–45)	43.5 (40–47)	.09
GGT (U/L)^‡^	48 (27.3–81.8)	39.5 (23–94.3)	.37
PLT (× 10^9^/L)^‡^	167 (121–226)	156.5 (108.8–197.3)	.11
INR^†^	0.98 (0.9–1.0)	1 (0.95–1.06)	.10
Total bile acid (μmol/L)^‡^	6.9 (4.1–11.4)	6.4 (3.4–10.8)	.73
Total cholesterol (mg/dl)^‡^	4 (3.6–4.6)	4 (3.4–4.4)	.24
Fibrosis stages			.99
S0	79	20	
S1	42	13	
S2	53	15	
S3	43	13	
S4	147	41	

^†^Data are mean ± standard deviation

^‡^Data are the median, with the interquartile range in parentheses

*ALT* alanine aminotransferase, *AST* aspartate aminotransferase, *ALB* albumin, *GGT* gamma-glutamyl transpeptidase, *PLT* platelet count, *INR* international normalized ratio

### Transfer learning vs non-transfer learning

**Fig. 3:**
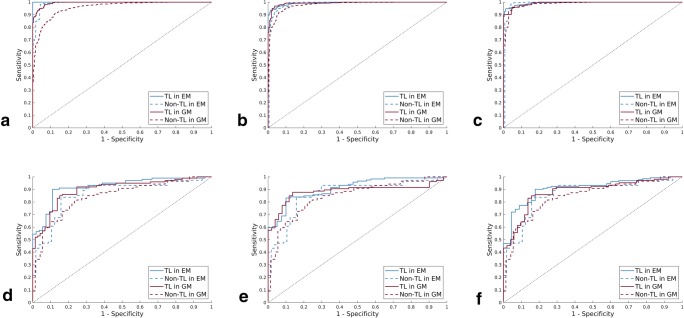
Comparison of ROC curves between TL and non-TL for the assessment of liver fibrosis stages in training and test cohort, respectively. **a**, **d** S0–S3 versus S4 in training and test cohort. **b**, **e** S0–S2 versus S3–S4 (≥ S3) in training and test cohort. **c**, **f** S0–S1 versus S2–S4 (≥ S2) in training and test cohort. TL, transfer learning; Non-TL, non-transfer learning

In the training cohort, TL in GM and EM demonstrated higher diagnostic accuracy (AUCs all ≥ 0.99) than non-TL for classifying S4, ≥ S3, and ≥ S2 (all *p* < .01) (Table 2). The AUCs of TL in GM reached 99.19%, 99.2%, and 99.42% for the three stratifications, respectively, which were 0.66%, 1.38%, and 3.95% higher than those of non-TL. The AUCs of TL in EM reached 99.37%, 99.34%, and 100% for the three stratifications, respectively, which were 0.59%, 0.82%, and 0.94% higher than those of non-TL. Because the EM had not only texture and brightness information but also color information, the AUCs in EM were slightly higher than those in GM (Fig. 3).

**Table 2:** The diagnostic performance of TL and non-TL in GM and EM

Stage and method	AUC	*p* value	Sensitivity (%)	Specificity (%)	PPV (%)	NPV (%)	LR+	LR−
Training cohort								
S4								
GM non-TL	0.957 (0.945–0.968)	< .001	91.0	88.4	88.8	89.9	7.2	0.1
TL	0.994 (0.945–0.968)		95.1	95.6	96.3	95.8	21.3	0.0
EM non-TL	0.991 (0.983–0.995)	.001	96.3	93.9	97.4	96.6	18.6	0.0
TL	1.0 (0.992–1.0)		100.0	100.0	100.0	100.0	/	0.0
≥ S3								
GM non-TL	0.978 (0.966–0.989)	.002	92.1	93.1	95.3	88.5	13.3	0.1
TL	0.992 (0.981–0.997)		96.9	95.0	97.1	95.0	19.0	0.0
EM non-TL	0.985 (0.977–0.990)	.002	96.5	89.1	95.0	92.7	8.7	0.0
TL	0.993 (0.983–0.998)		97.4	95.8	98.1	97.0	23.4	0.0
≥ S2								
GM non-TL	0.985 (0.976–0.991)	.001	93.9	95.2	97.58	87.27	19.5	0.1
TL	0.992 (0.9719–1.0)		95.7	94.4	97.50	95.04	22.3	0.0
EM non-TL	0.988 (0.981–0.991)	.001	92.8	96.9	98.33	84.77	20.9	0.1
TL	0.994 (0.981–0.998)		98.8	96.0	98.08	97.04	25.8	0.0
Test cohort								
S4								
GM non-TL	0.852 (0.785–0.901)	.002	81.6	75.3	79.8	77.5	3.3	0.2
TL	0.897 (0.831–0.940)		86.0	83.6	87.8	81.3	5.2	0.2
EM non-TL	0.862 (0.787–0.920)	.002	81.1	75.9	82.1	74.5	3.4	0.3
TL	0.921 (0.897–0.951)		89.1	87.0	92.8	81.3	6.9	0.1
≥ S3								
GM non-TL	0.843 (0.768–0.901)	.002	80.5	66.7	78.5	72.3	2.4	0.3
TL	0.885 (0.827–0.919)		87.7	84.3	92.1	76.8	5.6	0.2
EM non-TL	0.861 (0.785–0.916)	.002	83.6	82.5	91.8	76.7	5.1	0.1
TL	0.910 (0.853–0.952)		85.2	90.0	93.1	73.3	8.1	0.2
≥ S2								
GM non-TL	0.844 (0.769–0.902)	.001	70.0	74.1	76.2	67.2	2.7	0.4
TL	0.882 (0.825–0.918)		87.1	83.3	89.9	70.6	5.3	0.1
EM non-TL	0.867 (0.793–0.917)	.003	85.9	81.4	90.1	74.7	4.6	0.2
TL	0.907 (0.793–0.917)		87.9	83.2	90.2	76.0	5.5	0.2

Data in parentheses are 95% confidence intervals

*Non-TL* non-transfer learning, *TL* transfer learning, *GM* gray scale modality, *EM* elastogram modality, *NPV* negative predictive value, *PPV* positive predictive value, *LR*+ positive diagnostic likelihood ratio, *LR*− negative diagnostic likelihood ratio

In the test cohort, the AUCs of non-TL in GM were 3.84%, 4.19%, and 4.55% lower than the AUCs of TL (all *p* < .01). The AUCs of non-TL in EM were also 5.9%, 4.9%, and 4.0% lower than the AUCs of TL (all *p* < .01) (Fig. 3, Table 2).

In the single-modal experiments, the AUCs of non-TL were lower than that of TL. This is because ImageNet pretrained weights can be shared on the bottle layer, which not only solves the problem of insufficient data and overfitting but also extracts a number of excellent common features.

### Multimodalities vs single modalities

As the AUCs of TL were statistically higher than those of non-TL, we used the TL in the multimodal experiments.

**Fig. 4:**
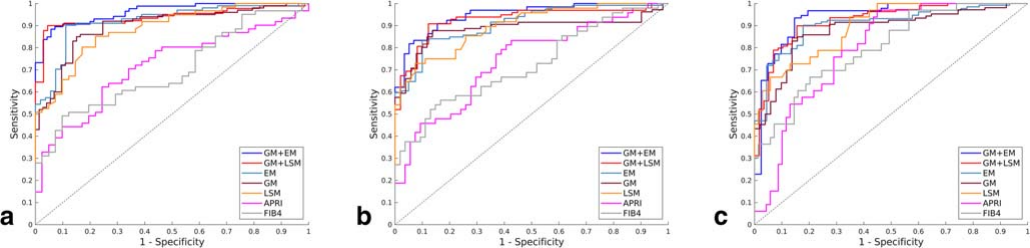
Comparison of AUCs between GM + EM, GM + LSM, EM, GM, LSM, APRI, and FIB-4 for the assessment of liver fibrosis stages in test cohorts. **a** S0–S3 versus S4 (S4); **b** S0–S2 versus S3–S4 (≥ S3); **c** S0–S1 versus S2–S4 (≥ S2). GM + EM, gray scale modality and elastogram modality; GM + LSM, gray scale modality and liver stiffness measurement; GM, gray scale modality; EM, elastogram modality; LSM, liver stiffness measurement

In the training cohort, EM demonstrated statistically higher AUCs than GM for the three stratifications (*p* < .001) (Table 3). In the test cohort, the AUCs of GM + LSM reached 92.0%, 92.7%, and 93.7% for diagnosing liver fibrosis ≥ S2, ≥ S3, and S4, respectively, which were significantly higher than the AUCs of GM and LSM alone (all *p* < .01). The AUCs of GM + EM were significantly higher than those of GM and EM alone (all *p* < .01) (Fig. 4). The sensitivity and specificity analyses also demonstrated that GM + LSM and GM + EM were universally better than GM, EM, and LSM alone (Table 3). GM + EM demonstrated the highest AUCs, reaching 93.0%, 93.2%, and 95.0% for the three stratifications, respectively, which were 1.0%, 0.5%, and 1.3% higher than GM + LSM (all *p* < .05).

**Table 3:** The diagnostic performance of EM, GM, LSM, APRI, and FIB-4 in evaluate liver fibrosis stages in training and test cohort

Stage and method	AUC	*p* value ^*^	*p* value ^**^	Sensitivity (%)	Specificity (%)	PPV (%)	NPV (%)	LR+	LR−
Training cohort									
S4									
APRI	0.715 (0.663–0.767)	< .001	< .001	65.9	66.3	56.8	75.0	1.9	0.5
FIB-4	0.690 (0.636–0.744)	< .001	< .001	69.1	61.2	55.8	72.4	1.9	0.6
LSM	0.926 (0.899–0.953)	< .001	< .001	84.8	91.2	79.8	93.3	5.8	0.1
GM	0.994 (0.984–0.999)	/	< .001	95.1	95.6	96.3	95.8	21.3	0.0
EM	1.0 (0.992–1.0)	/	/	100.0	100.0	100.0	100.0	/	0.0
≥ S3									
APRI	0.778 (0.730–0.827)	< .001	< .001	71.3	75.8	73.9	72.8	2.6	0.3
FIB-4	0.745 (0.695–0.795)	< .001	< .001	69.0	66.3	67.9	65.5	1.9	0.5
LSM	0.906 (0.876–0.937)	< .001	< .001	86.8	85.3	86.7	84.7	6.0	0.2
GM	0.992 (0.981–0.997)	/	< .001	96.9	95.0	97.1	95.0	19.0	0.0
EM	0.993 (0.983–0.998)	/	/	97.4	95.8	98.1	97.0	23.4	0.0
≥ S2									
APRI	0.781 (0.73–0.832)	< .001	< .001	75.2	73.3	84.8	58.2	2.8	0.4
FIB-4	0.729 (0.673–0.785)	< .001	< .001	62.8	73.3	79.7	54.4	2.0	0.4
LSM	0.906 (0.873–0.940)	< .001	< .001	82.6	87.7	89.5	75.6	4.2	0.2
GM	0.992 (0.972–1.0)	/	< .001	95.7	94.4	97.5	95.0	22.3	0.0
EM	0.994 (0.981–0.998)	/	/	98.8	96.0	98.1	97.0	25.8	0.0
Test cohort									
S4									
APRI	0.716 (0.617–0.815)	< .001	< .001	60.7	75.6	55.4	78.3	1.8	0.4
FIB-4	0.698 (0.598–0.798)	< .001	< .001	60.7	68.3	53.9	74.0	1.7	0.5
LSM	0.884 (0.821–0.947)	.003	< .001	83.6	78.0	72.7	84.5	4.0	0.3
GM	0.897 (0.831–0.940)	.002	< .001	86.0	83.6	87.8	81.3	5.2	0.2
EM	0.921 (0.897–0.951)	.01	.005	89.1	87.0	92.8	81.3	6.9	0.1
GM + LSM	0.937 (0.907–0.970)	/	.013	89.0	92.5	90.2	87.3	12.1	0.1
GM + EM	0.950 (0.917–0.972)	/	/	90.1	94.3	94.9	88.0	15.7	0.1
≥ S3									
APRI	0.741 (0.645–0.838)	.001	.001	58.3	72.2	65.0	64.3	1.7	0.5
FIB-4	0.721 (0.622–0.821)	< .001	< .001	58.3	68.5	64.9	62.2	1.6	0.5
LSM	0.898 (0.839–0.956)	.004	.001	83.3	74.1	80.3	74.5	3.9	0.3
GM	0.885 (0.827–0.919)	.003	.004	87.7	84.3	92.1	76.8	5.6	0.2
EM	0.910 (0.853–0.952)	.022	.004	85.2	90.0	93.1	73.3	8.1	0.2
GM + LSM	0.927 (0.893–0.958)	/	.016	87.8	85.8	90.5	78.2	8.2	0.1
GM + EM	0.932 (0.899–0.961)	/	/	89.9	87.9	90.7	80.3	8.1	0.2
≥ S2									
APRI	0.796 (0.711–0.881)	.001	< .001	63.6	73.9	81.0	53.9	2.0	0.4
FIB-4	0.801 (0.711–0.890)	.001	< .001	66.7	75.4	82.8	57.9	2.3	0.4
LSM	0.896 (0.834–0.957)	.004	.001	75.8	85.5	87.0	72.7	3.2	0.2
GM	0.882 (0.825-0.918)	.003	< .001	87.1	83.3	89.9	70.6	5.3	0.1
EM	0.907 (0.849–0.950)	.022	.007	87.9	83.2	90.2	76.0	5.5	0.2
GM + LSM	0.920 (0.886–0.951)	/	.019	88.0	88.2	92.5	87.3	6.6	0.1
GM + EM	0.930 (0.899–0.962)	/	/	90.0	87.8	94.2	77.6	7.2	0.1

Data in parentheses are 95% confidence intervals

^*^Compared with GM in training cohort and compared with GM + LSM in testing cohort

^**^Compared with EM in training cohort and compared with GM + EM in testing cohort

*GM* gray scale modality, *EM* elastogram modality, *LSM* liver stiffness measurement, *GM + EM*, gray scale modality and elastogram modality, *GM + LSM* gray scale modality and liver stiffness measurement, *NPV* negative predictive value, *PPV* positive predictive value, *LR*+ positive diagnostic likelihood ratio, *LR*− negative diagnostic likelihood ratio

### Transfer learning vs liver stiffness measurement and serum indexes

**Fig. 5:**
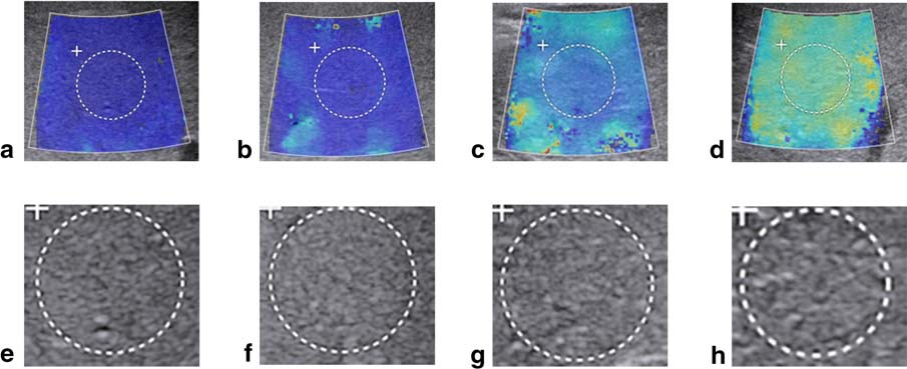
The demonstration of elastogram and gray scale modalities of different liver fibrosis stages. **a**, **e** Elastogram and gray scale modalities of S0~1. **b**, **f** Elastogram and gray scale modalities of S2. **c**, **g** Elastogram and gray scale modalities of S3. **d**, **h** Elastogram and gray scale modalities of S4

In the training cohort, the AUCs of GM and EM were significantly higher than those of LSM, APRI, and FIB-4 for identifying cirrhosis, fibrosis ≥ S3, and ≥ S2 (all *p* < .0001). In the test cohort, the multimodalities (GM + LSM and GM + EM) and single modalities (GM and EM alone) all demonstrated higher diagnostic accuracy than LSM and serum indexes for classifying S4, fibrosis ≥ S3, and ≥ S2, and differences in the AUCs were all significant (*p* < .01) (Table 3). Figure 5 demenstrates the various stages of liver fibrosis with both gray scale and elastogram modality.

## Discussion

The accurate and non-invasive classification of liver fibrosis is of crucial importance in clinical practice. Deep learning system for staging liver fibrosis using CT images has been reported recently and showed good performance [29, 30]. US is a more common and non-invasive imaging modality for routine examination, and there have been few reports of deep learning used in the analysis of US images. In this study, we analyzed not only gray scale images but also elastogram images of 2D SWE in CHB-infected patients with transfer learning for the classification of liver fibrosis. Thus far, there have been no reports on the diagnostic value of transfer learning in combination of GM and EM for assessing liver fibrosis stages.

Gray scale US images contain original information, such as the reflection and scattering of fine structures in the liver parenchyma, which is associated with the accumulation of collagen fibers, a loss of portal vein wall definition, and irregularity of hepatic vein margins, all indicative of the process of liver fibrosis. Coarse hepatic echotexture and mildly increased echogenicity of the liver parenchyma are common in cirrhosis. The assessment of these findings is subjective, however, with poor inter- and intraobserver agreement, and the findings also largely depend on the equipment used [31]. Furthermore, these indicators are seen mainly in cirrhosis and are less frequent in the early stages of fibrosis. Therefore, a quantitative and objective method for analyzing gray scale US images might be valuable. In the study, we provide an objective method, transfer learning, to explore the valuable information of gray scale US images.

Histopathologically, hepatic fibrosis is a consequence of the excessive accumulation of extracellular matrix components in the liver. This process is caused by a wound healing response to persistent liver damage, inducing hepatic stellate cell activation, high alpha smooth muscle actin production, and collagen type I and III secretion, and can progress to cirrhosis [32]. The stiffness of the liver parenchyma increases with the progression of liver fibrosis, which can be reflected by LSM and the color-coded elastograms of 2D SWE.

In the case of medical image analysis, the implementation of TL techniques has been reported in several papers [33–35]. Banerjee et al [36] adapted a TL approach in which the pretrained AlexNet model was fine-tuned on fused multimodal MR scans for rhabdomyosarcoma soft tissue sarcoma classification. In this study, we used TL to objectively assess gray scale and elastogram images, which demonstrated good performance than non-TL. These results showed that the weights learned by using a large number of natural images could be better applied to medical images through fine-tuning.

We performed an innovative multimodal analysis, including GM + LSM and GM + EM. In the GM + LSM analysis, based on the characteristics and clinical analysis of the confidence function, we constructed a confidence function of the mathematically significant LSM. Meanwhile, we creatively combined GM and EM, automatic classifier learning was achieved through three fully connected layers. The multimodal GM + EM and GM + LSM methods demonstrated superior performance compared to the single-modal methods, indicating that the multimodalities carried more diagnostic information.

There have been some reports on traditional machine learning and deep learning methods for diagnosing CLD. Gatos et al [37] reported a multicenter study of 126 patients with 2D SWE images, from which they extracted 35 hard-coded radiomic features; the AUC reached 0.87 for the proposed machine learning method. Kayaaltı et al [15] obtained a comprehensive set of texture features from CT images which were classified using two methods, namely, support vector machines and k-nearest neighbors. Kun Wang et al [18] performed a study evaluating the value of deep learning radiomics of shear wave elastography (DLRE) in staging liver fibrosis in CHB-infected patients and reported that DLRE showed the best overall performance compared with LSM and serum indexes. There are some differences between their study and our study. Their model referred to the information of EM rather than GM and did not develop a more comprehensive integration of the two modalities. Furthermore, we concluded that the TL method converges faster than the non-TL method. Based on the published literatures, we summarized some reported methods and performance of traditional machine learning and deep learning on analyzing medical images to assess liver fibrosis in Table S1 in the supplementary material.

There were some limitations in our study. First, the distribution of patients among fibrosis stages, particularly S4, was uneven. This was mainly because of the large proportion of patients with hepatocellular carcinoma and cirrhosis among those who underwent partial hepatectomy. Second, the number of patients in our study was limited; thus, a multicenter validation and prospective studies should be performed to evaluate the value of TL in GM and EM. Third, we will study how to extract better features suitable for the current domain from across fields and study a more generalized model for liver fibrosis staging. Fourth, the gray scale and elastogram ultrasound images are susceptible to reconstruction and processing algorithms, which may affect the diagnosis performance of the method of a deep convolutional neural network by a transfer learning modal.

In conclusion, liver fibrosis can be staged by transfer learning modal with better performance than non-transfer learning, and the combination of gray scale modality and elastogram modality was the most accurate prediction model compared with the combination of gray scale modality and liver stiffness measurement, gray scale modality, elastogram modality, and liver stiffness measurement alone, and serum liver fibrosis indexes. These results indicate that transfer learning in gray scale and elastogram modality is a promising method with potential for application in clinical liver fibrosis staging, and further multicenter and large-scale studies should be performed to improve and verify the model.

## Electronic supplementary material

ESM 1 (DOCX 244 kb)

## Abbreviations

2D SWE: Two-dimensional shear wave elastography
APRI: Aspartate aminotransferase to platelet ratio index
AUC: Area under the receiver operating characteristic curve
CHB: Chronic hepatitis B
CLD: Chronic liver disease
EM: Elastogram modality
FIB-4: Fibrosis index based on the four factors
GM: Gray scale modality
IQR: Interquartile range
LR−: Negative likelihood ratios
LR+: Positive likelihood ratios
LSM: Liver stiffness measurement
Non-TL: Non-transfer learning
NPV: Negative predictive values
PPV: Positive predictive values
TL: Transfer learning
