# Supplementary material for: Transfer learning radiomics based on multimodal ultrasound imaging for staging liver fibrosis
Source: Eur Radiol. 2020 Jan 21;30(5):2973–83. doi: 10.1007/s00330-019-06595-w (PMC7160214; doi:10.1007/s00330-019-06595-w)
Supplement: Supplementary file 1 — (DOCX 244 kb) [file 330_2019_6595_MOESM1_ESM.docx]

**Transfer Learning Radiomics Based on Multimodal Ultrasound Imaging for Staging Liver Fibrosis**

**Supplementary material**

**Transfer Learning (TL)**

The medical dataset was used to fine-tune Inception-V3. The ROI images needed to be unified in size to 299×299×3 pixels. The stochastic gradient descent optimizer (Equation. 1) with momentum was employed to train the Inception-V3 network, with a batch size of 64 for both training and validation.

***
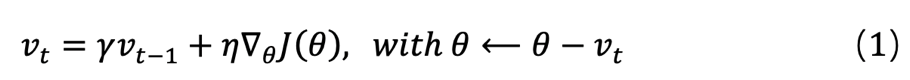
***

***where*** $v_{t}$ is the current velocity vector, J(θ) is the objective function parameterized by a model’s parameters θ, $\nabla_{\theta}J$(.) represents the gradient of the objective function, η is the learning rate, and γ denotes a ﬁxed weight such that γ ∈ [0, 1]. The momentum helps accelerate the SGD in the relevant direction and mitigates oscillations. It performs this by adding a fraction of the update vector of the past time step to the current update vector. A learning rate and momentum weight of 0.0001 and 0.9, respectively, were found to be suitable.

We trained the Inception-V3 model by non-TL for comparative experiments. We randomly initialized weights W drawn from a normal distribution, as follows: *N* (*μ*=0, *σ*^2=0.05).

**Grayscale Modality + Liver Stiffness Measurement (GM+LSM)**

The confidence coefficient of each image and the accuracy of the model can be obtained from the previous grayscale experiment. However, the confidence coefficient for each image cannot be obtained by 2D SWE. Hence, based on the cutoff value, a confidence function, as shown in equation 2, is constructed in this paper.


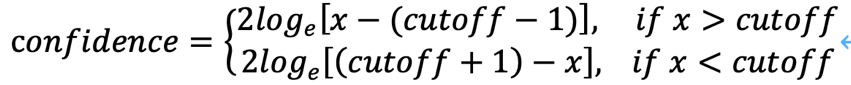
 (2)

Here, *x* is the LSM of a certain image, and cutoff is the cutoff point value for a certain classification. As shown in the equation 2 and the function graph (Figure S1), the idea of the construction of this function is that the closer the LSM is to the cutoff, the more likely it is to be misclassified, leading to reduced confidence. Conversely, the farther away the LSM is from the cutoff, the higher the confidence.


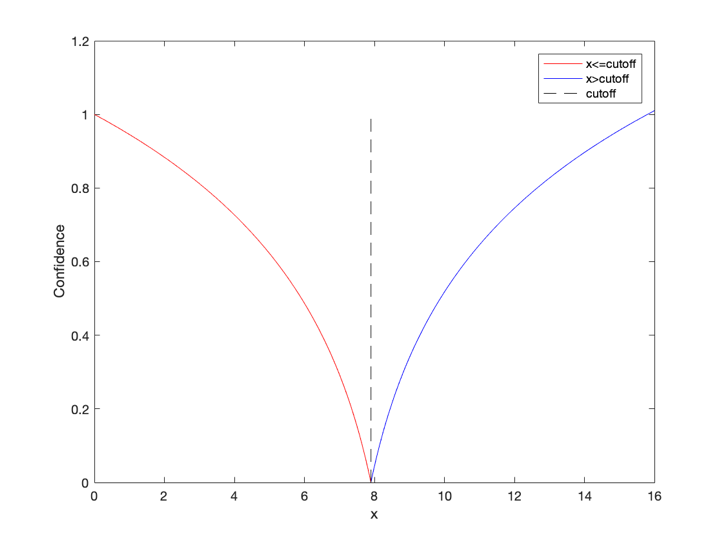


Figure S1. Confidence function graph

After obtaining the confidence of 2D SWE, we combined it with the confidence of grayscale imaging as features input by logistic regression. Let the grayscale image confidence be x1, the 2D SWE confidence be x2, and the input of the sigmoid function be z. Therefore, z=θ0+θ1*x1+θ2*x2; here, θ0 is a bias, and θ1 and θ2 are the best parameters that we want to fit. We then implemented mini batch gradient descent to find optimal parameter for classification. If the number of iterations reaches a given value or the algorithm is in an allowable error range, they satisfy the conditions to stop iterations. The initial values of θ1 and θ2 are set to the accuracy of the grayscale image and the 2D-SWE. We stop the iteration when the number of iterations is 500. The gradient step size is 0.001.

**Grayscale Modality + Elastogram Modality (GM+EM)**

After extracting the 2048-dimensional features by bottleneck of the two modal images separately, we concatenate features of two modals into 4096-dimentional ones and use 3 fully connected layers as a classifier. To avoid overﬁtting, we adopt the dropout strategy. Figure S2 describes our proposed workflow for the GM+EM.

In this paper, we used focal loss to solve the problem of data category imbalance and easy sample overwhelming to improve the accuracy of model training. The focal loss approach is shown in equation 3.


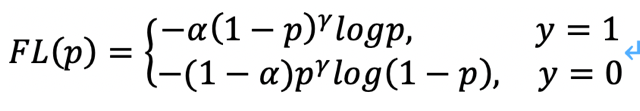
 (3)

Here, *p* is the predicted probability. Focal Loss first solved the problem of data imbalance, which is multiplied by the weight in the loss calculation. When the class was 1, we multiplied by weight $\alpha=0.25$, otherwise, we multiplied by weight 1-$\alpha$. In addition, we took $\gamma=2$ to reduce the loss of easily classified samples and focus more on the ones that were more easily misclassified.


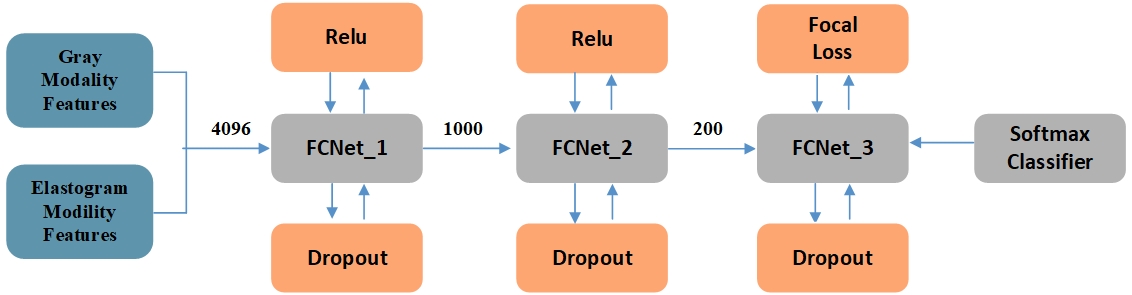


Figure S2. The architecture of GM+EM fully connected neural network (FCNet).

Table S1. Reported methods and performance of traditional machine learning and deep learning for assessing chronic liver disease

| Author | Year of Study | Method | Type of Medical Image | Database Size | AUC | Accuracy |
| --- | --- | --- | --- | --- | --- | --- |
| Lee JH [1] | 2019 | Develop a deep convolutional neural network | Grayscale ultrasound images | 4547 patients | 0.857 for F4 | / |
| Gao S [2] | 2014 | Texture analysis | Grayscale ultrasound images | 37 patients | / | 100%, 90%, 70%, 90% and 100% for S0~S4 |
| Kayaaltı Ö [3] | 2014 | Texture analysis, support vector machines (SVM) and k-nearest neighbors (k-NN) | Venous phase CT images | 116 patients | / | 93.17% using k-NN  94% using SVM |
| Wang K [4] | 2018 | Develop deep learning radiomics of elastography | Ultrasound elastography images | 398 patients | 0.97 for F4  0.98 for ≥F3  0.85 for ≥F2 | / |
| Yasaka K [5] | 2018 | Develop a deep convolutional neural network | Portal phase CT images | 286 patients | 0.74 for ≥F2  0.76 for ≥F3  0.73 or F4 | / |
| Choi KJ [6] | 2018 | Develop a deep learning system | Portal venous phase CT images | 7461 patients | 0.96 for ≥F2  0.97 for ≥F3  0.95 for F4 | 79.4% |
| Gatos I [7] | 2017 | Support vector machine classiﬁcation algorithm | Ultrasound elastography images | 126 patients | 0.87 for ≥F1 | 87.3% |
| Acharya UR [8] | 2018 | 2D contourlet transform and kernel discriminant analysis | Grayscale ultrasound images | 236 patients | / | 91.46% |
| Yu Y [9] | 2018 | Transfer learning with AlexNet Convolutional Neural | Second Harmonic Generation (SHG) microscopy images | 25 rats | 0.85–0.95 | / |
| Byra M [10] | 2018 | Inception-ResNet-v2 deep convolutional neural network pre-trained on the ImageNet dataset | Grayscale ultrasound images | 55 patients | 0.977 | 96.3% |
| Yeh WC [11] | 2003 | Gray level concurrence, nonseparable wavelet transform and support vector machine | Grayscale ultrasound images | 20 fresh postsurgical human liver samples | / | 91% for 2 classes (F0-3, F4-5) |
| Khvostikov A [12] | 2017 | Anisotropic diffusion method and a total variation method for ultrasound image speckle filtering | Grayscale ultrasound images | 60 patients | / | 90% for 3 classes (F0, F1–F3, F4) |

[1] Lee J H, Joo I, Kang T W, et al (2019) Deep learning with ultrasonography: automated classification of liver fibrosis using a deep convolutional neural network[J]. European radiology, 1-10

[2] Gao S, Peng Y, Guo H, et al (2014) Texture analysis and classification of ultrasound liver images. Biomed Mater Eng 1209–1216

[3] Kayaaltı Ö, Aksebzeci BH, Karahan İÖ, et al (2014) Liver fibrosis staging using CT image texture analysis and soft computing. Appl Soft Comput 25:399–413

[4] Wang K, Lu X, Zhou H, et al (2018) Deep learning Radiomics of shear wave elastography significantly improved diagnostic performance for assessing liver fibrosis in chronic hepatitis B: a prospective multicentre study. Gut 68:729–741

[5] Yasaka K, Akai H, Kunimatsu A, et al (2018) Deep learning for staging liver fibrosis on CT: a pilot study. Eur Radiol 28:4578–4585

[6] Choi KJ, Jang JK, Lee SS, et al (2018) Development and validation of a deep learning system for staging liver fibrosis by using contrast agent–enhanced CT images in the liver. Radiology 289:688–697

[7] Gatos I, Tsantis S, Spiliopoulos S, et al (2017) A machine-learning algorithm toward color analysis for chronic liver disease classification, employing ultrasound shear wave elastography. Ultrasound Med Biol 43:1797–1810

[8] Acharya U R, Raghavendra U, Koh J E W, et al (2018) Automated detection and classification of liver fibrosis stages using contourlet transform and nonlinear features[J]. Computer methods and programs in biomedicine, 166: 91-98

[9] Yu Y, Wang J, Ng C W, et al (2018) Deep learning enables automated scoring of liver fibrosis stages[J]. Scientific reports, 8: 16016

[10] Byra M, Styczynski G, Szmigielski C, et al (2018) Transfer learning with deep convolutional neural network for liver steatosis assessment in ultrasound images[J]. International journal of computer assisted radiology and surgery, 13: 1895-1903

[11] Yeh W C, Huang S W, Li P C (2003) Liver fibrosis grade classification with B-mode ultrasound[J]. Ultrasound in medicine & biology, 29: 1229-1235

[12] Khvostikov A, Krylov A, Kamalov J, et al (2017) Ultrasound despeckling by anisotropic diffusion and total variation methods for liver fibrosis diagnostics[J]. Signal Processing: Image Communication, 59: 3-11.
